# Could statistical potential models achieve comparable or better performance than deep learning models?

**DOI:** 10.1093/bib/bbag088

**Published:** 2026-03-02

**Authors:** Zhihao Wang, Sheng Wang, Jingjing Guo, Yuguang Mu, Xiangdong Liu, Liangzhen Zheng, Weifeng Li

**Affiliations:** School of Physics, Shandong University, 27 Shanda Nan Road, 250100 Jinan, Shandong Province, China; Shanghai Zelixir Biotech Co. Ltd., 298 Xiangke Road, 201210 Shanghai, China; Centre for Artificial Intelligence Driven Drug Discovery, Faculty of Applied Science, Macao Polytechnic University, R. de Luís Gonzaga Gomes, 999078 Macao, China; School of Biological Sciences, Nanyang Technological University, 50 Nanyang Avenue, 639798, Singapore; School of Physics, Shandong University, 27 Shanda Nan Road, 250100 Jinan, Shandong Province, China; Shanghai Zelixir Biotech Co. Ltd., 298 Xiangke Road, 201210 Shanghai, China; School of Physics, Shandong University, 27 Shanda Nan Road, 250100 Jinan, Shandong Province, China

**Keywords:** statistical potential, protein–ligand interaction, scoring function

## Abstract

Accurately predicting protein–ligand interactions is vital for structure-based drug discovery. Although deep learning (DL) models have shown strong performance, the potential of traditional statistical potentials under data-limited conditions remains underexplored. Here, we systematically assess several statistical potential models in docking and virtual screening. We find that docking benefits from distance-dependent pairwise atom–atom potentials with clear physical meanings, while screening relies more on orientation-dependent atom–residue potentials that capture local chemical environments. Based on these findings, we propose HybridSP, a hybrid potential combining distance-dependent atom–atom, atom–residue, and orientation-dependent atom–residue terms. An affinity-weighted scheme is applied to correct biases in statistical distributions. On the CASF-2016 benchmark, HybridSP achieves a 91.6% docking success rate and an enrichment factor of 29.35 at the top 1%, rivaling and even surpassing state-of-the-art DL models. Its strong screening ability is further validated on directory of useful decoys-enhanced and directory of useful decoys-adjusted. These results demonstrate that well-designed statistical potentials can achieve high performance and interpretability without complex DL architectures, offering an efficient alternative for scoring function design. The models are available at: https://github.com/zelixirSH/HybridSP.git.

## Introduction

For decades, researchers have been striving to elucidate the 3D structures of biomacromolecules, as structure determines function [1]. A clear understanding of these structures not only deepens our knowledge of biological processes but also enables rational drug design, disease mechanism studies, and biotechnological applications. With the aid of experimental techniques such as X-ray diffraction, nuclear magnetic resonance, and cryo-electron microscopy (CryoEM), nearly 240 000 biomolecular structures have been determined [2–4], providing fundamental insights into molecular mechanisms and greatly advancing modern biomedicine [5–7]. However, these experimental approaches are often costly, time-consuming, and not suitable for large-scale or high-throughput studies.

To overcome these limitations, computational approaches based on physical principles and expert knowledge have emerged [8, 9]. These methods offer faster structural insights and have guided experimental efforts. Nevertheless, quantum mechanics-based simulations remain computationally demanding, force-field methods often lack accuracy, and empirical scoring functions may overlook the full complexity of biomolecular interactions [10–12].

The rise of deep learning (DL) has transformed structural biology. Models such as AlphaFold and RoseTTAFold achieve near-experimental accuracy in protein structure prediction [13–17]. AlphaFold3 further extends this capability to multi-component complexes containing proteins, nucleic acids, small molecules, and ions [15]. Despite these successes, recent benchmarks reveal that DL-based models, while powerful, often lack explicit physical and chemical priors and generalize poorly to unseen protein–ligand systems [18].

A notable example is seen in CASP15, where AIchemy_RNA2, a human expert-guided model, outperformed DL-only methods in RNA structure prediction [15, 19, 20]. Its success relied on a classical energy-based scoring function derived from the BRiQ statistical potential [21, 22]. This finding raises a crucial question: under data-limited conditions, can statistical potential methods perform as well as, or even better than, DL models?

Motivated by this question, we focus on protein–ligand interactions, a key component of structure-based drug design. Accurately modeling these interactions accelerates the identification of potential lead compounds and significantly improves drug discovery efficiency [23]. Yet, it remains difficult to achieve balanced performance in both docking and virtual screening tasks [24, 25]. Docking, which focuses on identifying the optimal binding pose, is generally easier—many scoring functions achieve success rates $\sim $80% on CASF-2016 [26]. However, their screening performance, which measures the ability to distinguish true binders from decoys, remains unsatisfactory [26].

Early DL-based scoring models also suffered from this imbalance [27, 28], but with the introduction of graph neural networks and Transformer architectures, both docking and screening accuracy have improved significantly [29]. State-of-the-art DL models now reach docking success rates >90% and enrichment factors (EFs) two to three times higher than traditional approaches [25, 30]. In contrast, most knowledge-based statistical potentials achieve docking success rates <85% and EFs below 7, highlighting their limited capacity to generalize across diverse binding interactions.

In this work, we propose several novel statistical potential models to explore the relationship between statistical interaction patterns and model performance in docking and screening tasks. Our results reveal that the two tasks emphasize distinct interaction types: distance-dependent atom–atom potentials with clear physical interpretation enhance docking, whereas orientation-dependent atom–residue potentials are more critical for screening. Building on these findings, we developed HybridSP, a hybrid statistical potential integrating atom–atom distance, atom–residue distance, and atom–residue orientation potentials. HybridSP achieves a 91.6% docking success rate and an EF of 29.35 at the top 1% on CASF-2016, rivaling or surpassing the best DL-based scoring functions. On the DUD-E dataset, it reaches an EF of 40.27 within the top 0.5%, further confirming its strong ability to identify true binders. We attribute this competitive performance to the explicit incorporation of physically meaningful interaction patterns, which promotes robustness and generalization, particularly in scenarios where training data are limited or interaction geometries deviate from learned distributions. These results demonstrate that carefully designed statistical potentials can approach or even exceed DL performance under data-limited conditions, offering a more interpretable and physically grounded framework for structure-based drug discovery.

## Materials and methods

### Computational methods

To obtain a more comprehensive understanding of how different statistical patterns affect the performance of knowledge-based scoring functions, we propose several statistical potential models. The details of each model were clarified in Supplementary materials.

#### Distance-dependent pairwise atom–residue statistical potential

Distance-dependent pairwise statistical potentials describe the interaction energy associated with the distance $r$ between protein and ligand components. Following the statistical framework proposed in DrugScore2018, where potentials are derived from pair distribution functions, we constructed a modified model that considers residue-specific atomic environments. The pair distribution function between ligand atom $i$ and protein atom $j$ is calculated as:


(1) \begin{align*} & g_{i,j}(r)=\rho_{i,j}(r)/\rho_{i,j,\mathrm{bulk}}, \end{align*}

(2) \begin{align*} & \rho_{i,j}(r)=N_{i,j}(r)/4\pi r^{2}\mathrm{d}r, \end{align*}

(3) \begin{align*} & \rho_{i,j,\mathrm{bulk}}={\sum_{r=r_{0}}^{r_{c}}3N_{i,j}(r)}/(4\pi (r_{c}^{3}- r_{0}^{3})), \end{align*}

where $\rho _{i,j}(r)$ and $\rho _{i,j,\mathrm{bulk}}$ denote the local and bulk densities, respectively. The lower and upper limits of the interaction range are set to $r_{0}=2$ Å and $r_{c}=6$ Å, with an interval of $\mathrm{d}r=0.2$ Å. The reference state is defined as the average distribution of all atom–pair combinations:


(4) \begin{align*}& g(r)=\frac{\sum_{i}\sum_{j}g_{i,j}(r)}{N_{\mathrm{L}}\times N_{\mathrm{R}}},\end{align*}

and the pseudo free energy difference at distance $r$ is obtained via the inverse Boltzmann relation:


(5) \begin{align*}& \Delta W_{i,j}(r)=-k_{B}T\ln{\frac{g_{i,j}(r)}{g(r)}}.\end{align*}

By summing over all protein and ligand atom pairs, the total potential energy of the complex is:


(6) \begin{align*}& \Delta W=-\sum_{i,j,r}\ln{\frac{g_{i,j}(r)}{g(r)}}.\end{align*}

While traditional atom–atom potentials such as DrugScore and ITScoreAff effectively capture general distance-dependent interactions, they treat identical atom types in different residues as equivalent, neglecting residue-specific effects. Since the same atom type may exhibit distinct physicochemical environments in different residues, we introduce a distance-dependent pairwise atom–residue statistical potential, termed DrugResidue. In this model, protein atoms are classified into 100 residue-dependent types, where each residue contributes two backbone and three side-chain heavy atoms (four for glycine), together with an additional category for undefined or non-residue atoms such as metals, halogens, and cofactors. Ligand atoms follow 23 Sybyl atom types. This residue-aware redefinition allows the potential to better capture context-dependent interaction patterns and improve the accuracy of statistical modeling at the protein–ligand interface.

#### Affinity weighted statistical model

As discussed in the “Discussion” section, the aim of a knowledge-based scoring function is to learn as faithful a representation of the true statistical distribution as possible. If the training set contains a large number of low-affinity complexes, the model may fail to interpret the free-energy profile. To address this, we therefore use the receptor–ligand binding affinity as a key weighting factor. Specifically, for any complex with affinity $A$, we assign its contact-count weight $p$ as


(7) \begin{align*}& p=e^{(A-A_{\mathrm{mean}})/A_{\mathrm{mean}}},\end{align*}

where $A_{\mathrm{mean}}$ denotes the mean binding affinity across all training samples. Variants of DrugScore or DrugResidue that incorporate affinity-weighted schemes are denoted with a subscript W to indicate the use of weighted training.

### Datasets

#### Training set

All of the proposed statistical models, including the re-derived DrugScore$^{\mathrm{Re}}$, were trained on the protein–ligand complex dataset from PDBbind 2020 [31]. Structures with a resolution larger than $2.5$ Å were excluded from the training set. In addition, entries sharing the same PDB code with the test sets were removed to prevent data leakage and evaluation bias. This results in 15 145 structures in total.

#### Test sets

The Comparative Assessment of Scoring Functions 2016 (CASF-2016) benchmark, comprising 57 targets with 5 known ligands each, is widely used to evaluate scoring function performance across four key tasks: scoring, ranking, docking, and screening [26]. For the docking task, we additionally employed the PoseBusters and Unbias-v2019 datasets. PoseBusters is a toolkit for assessing pose plausibility [32]; here, we used it solely to calculate the docking success rate (RMSD $<2$ Å), as all poses were generated with AutoDock Vina. The Unbias-v2019 dataset provides a more rigorous, bias-minimized benchmark for docking [33]. To evaluate screening power, we used the Directory of Useful Decoys-Enhanced (DUD-E) dataset [25, 34] and its adjusted version DUD-AD [35]. We further constructed a P450 dataset (eight targets, active/inactive ratio 1:200); construction details are in the Supplementary Materials. Finally, we assessed HybridSP on a free energy perturbation (FEP) dataset compiled by Kuhn *et al.* [36], comparing its ability to reproduce experimental binding affinities against established FEP methods (FEP+ [37] and AMBER [38]).

#### Scoring functions for comparison

In this study, we compared the predictive performance of various statistical potential models with representative DL-based approaches, whose key characteristics are summarized in Table 1. To ensure a fair and meaningful comparison, we selected DL baselines that have been reported to achieve state-of-the-art or near–state-of-the-art performance on one or more relevant benchmarks [24, 25, 30, 39, 40]. All models were evaluated under consistent protocols using the same datasets and metrics.

**Table 1:** Scoring functions and their characteristics

Scoring functions	Model type	Input	Prediction target	Training dataset
HybridSP	Statistical potential	Atom–atom contact; Atom–residue contact	Binding energy	PDBbind-v2020
DrugScore$^{\mathrm{Re}}$	Statistical potential	Atom–atom contact	Binding energy	PDBbind-v2020
DrugResidue	Statistical potential	Atom–residue contact	Binding energy	PDBbind-v2020
KORP-PL [41]	Statistical potential	Atom–residue contact	Binding energy	PDBbind-v2016
ITScoreAff [42]	Statistical potential	Atom–atom contact	Binding energy	PDBbind-v2020
GenScore [25]	Gated graph convolutional network	Graph representation	Distance likelihood	PDBbind-v2020
RTMScore [30]	Graph transformer	Graph representation	Distance likelihood	PDBbind-v2020
IGModel [24]	Graph attention network	Graph representation	RMSD; binding affinity	PDBbind-v2019
DeepRMSD [39]	Multi-layer perceptron	Distance-based feature vector	RMSD	PDBbind-v2019

## Results

### The model of HybridSP

Hybrid statistical potential (HybridSP) is a knowledge-based scoring function that integrates three complementary distance-dependent statistical potentials: DrugResidue$_{\mathrm{W}}$, ITScoreAff [42], and KORP-PL [41]. Each model is designed to capture distinct aspects of protein–ligand interactions.

ITScoreAff, developed by the Huang group, employs an iterative distance-dependent atom–atom potential to better discriminate native complexes from decoys, thereby capturing realistic pairwise interaction patterns. KORP-PL introduces orientation-dependent potentials between ligand atoms and receptor backbones, providing a 3D interaction surface for directional recognition.

Our proposed DrugResidue$_{\mathrm{W}}$ serves as a theoretical refinement and extension of the DrugScore framework. Building upon the same statistical foundation, it introduces residue-dependent protein atom types to account for environmental variations among identical atom species in different residues, while simultaneously incorporating an affinity-weighted training scheme to mitigate the influence of low-affinity complexes. The theoretical derivation and atom-type definitions are detailed in Section “Distance-dependent pairwise atom-residue statistical potential.” In summary, DrugResidue$_{\mathrm{W}}$ represents a distance-dependent pairwise atom–residue statistical potential enhanced by affinity weighting.

**Figure 1:**
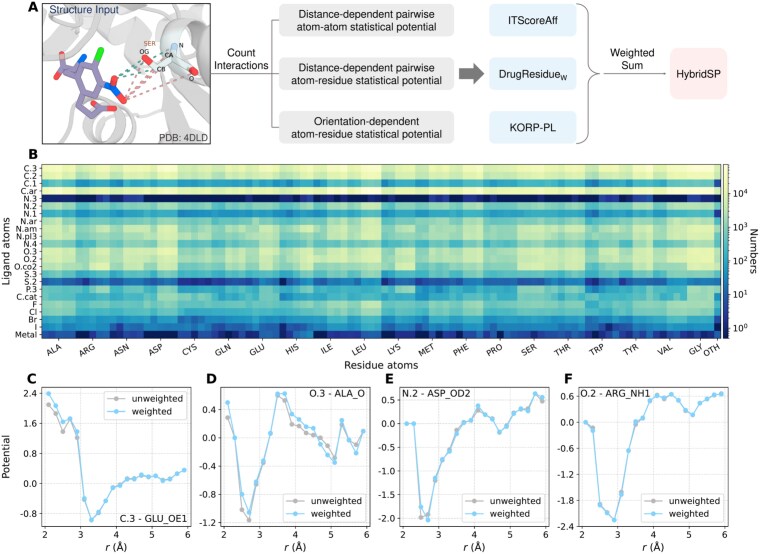
Overview of HybridSP and the statistical results. (A) The workflow of HybridSP model. (B) Number of pair interactions of the respective atom types of ligand and receptor. (C–F) Statistical potentials of different pair interactions.

As illustrated in Fig. 1A, HybridSP computes the statistical potentials of each model independently and combines them through a weighted summation:


(8) \begin{align*}& \mathrm{HybridSP} = a \times \mathrm{DrugResidue}_{\mathrm{W}} + b\times \mathrm{ITScoreAff} + c \times \mathrm{KORP-PL}.\end{align*}

The weighting coefficients $(a, b, c)$ are tuned for different applications. For docking-oriented scoring (HybridSP_dk_), the weights are $a=0.4$, $b=0.5$, and $c=0.1$. For screening-oriented scoring (HybridSP_scr_), the weights are $a=0.6$, $b=0.2$, and $c=0.2$. For the balanced model (HybridSP_bl_), designed to achieve robust performance in both docking and screening, the weights are $a=0.5$, $b=0.3$, and $c=0.2$.

Although the three components differ in formulation, they share a consistent distance-dependent statistical basis. Combining them through weighted summation introduces no conceptual inconsistency for two reasons. First, solvation effects are inherently many-body interactions that cannot be captured by simple pairwise decomposition [43]; integrating multiple statistical potentials helps approximate these collective effects. Second, the weighting procedure serves to normalize possible redundancies among distance-dependent interactions, similar to the approach adopted in the Vina scoring function [44].

### Statistical results of DrugResidue$_{\mathrm{W}}$

We analyzed the number of ligand–receptor atomic contacts within $2$–$6$ Å (Fig. 1B) [45]. Ligand atoms such as C, N, and O frequently interact with receptor atoms, forming clear potential energy curves. In contrast, atoms like N.3 and S.2 show fewer contacts, suggesting they are mostly located beyond $6$ Å from the pocket residues.

The theoretical basis of statistical potentials resembles that of free energy calculations, both relying on the Boltzmann relation to convert probability distributions into energy functions [46, 47]. Hence, accurate sampling of representative states is essential. In physics-based free energy simulations, insufficient sampling of high-energy states is often addressed through enhanced sampling and reweighting techniques [48–51]. Statistical potentials, however, are derived from crystal structures that mainly capture low-energy conformations with different binding affinities [26]. We hypothesize that low-affinity complexes include more high-energy contacts, while high-affinity complexes contain more favorable ones. Our previous work [39] showed that including LJ-like pair potentials in DL models acts as weighted potentials and improves docking accuracy. Therefore, treating all atomic pairs equally may bias the derived distributions. To mitigate this, we propose an affinity-weighted statistical scheme (see Materials and methods), weighting each atomic pair by its complex’s binding affinity.

Figure 1C–F compares weighted and unweighted distance-dependent potentials. For C.3–GLU_OE1 and O.3–ALA_O pairs, clear deviations appear mainly in high-energy regions, indicating sampling bias. The affinity-weighted approach smooths these curves, yielding more reliable energy profiles. For N–O pairs, both curves align well, likely because hydrogen bonds dominate high-affinity complexes. The potential curves also reveal interaction features: the $3.3$ Å well in C.3–GLU_OE1 may indicate a weak C–O hydrogen bond, while the $<3$ Å and $5$ Å wells in O–N pairs correspond to hydrogen bonds and possible salt bridges.

### Docking performance of HybridSP on different benchmarks

**Figure 2:**
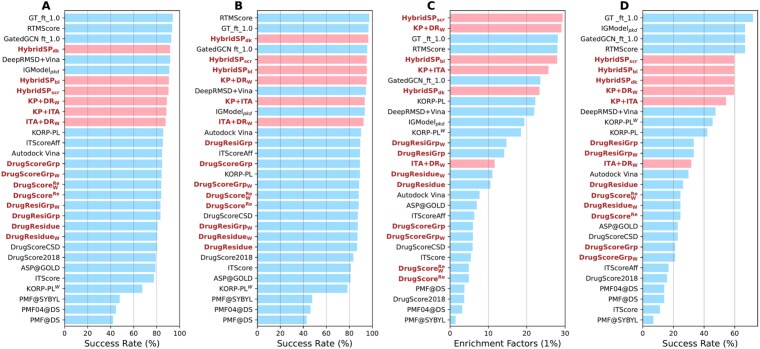
Docking and screening performance on CASF-2016 benchmark. Top 1 success rate (RMSD $<2$ Å) on docking benchmark with (A) and without (B) crystal poses included in the test set. ITA stands for ITScoreAff, DR$_{\mathrm{W}}$ refers to DrugResidue$_{\mathrm{W}}$, and KP represents KORP-PL. (C) Screening performance represented by the EF (1%). (D) Top 1% success rate on CASF-2016 screening task.

In traditional molecular docking, the protein binding pocket is typically treated as rigid or semi-flexible, while the small molecule is allowed to flexibly sample poses within it using docking software. These poses are then scored and ranked. Template-based modeling is another classical approach [52]. More recently, methods like AlphaFold3 have enabled fully flexible joint structure prediction through diffusion-based generative strategies [15, 53]. In this work, we adopt the traditional “dock-then-score” framework to evaluate docking performance. Figure 2A and B shows the success rates for identifying the top-scoring poses on the CASF-2016 benchmark, evaluated with and without the crystal pose, respectively. The methods include statistical potentials, empirical scoring functions (AutoDock Vina [44]), and several recent top-performing DL models [24, 25, 30, 39]. Scoring functions newly proposed in this study are highlighted in bold.

Results indicate that, except for PMF and its variants, traditional scoring functions generally achieve docking success rates of 80%–85%, whereas DL-based methods often exceed 90%. Our re-derived and improved models based on the DrugScore framework show consistent improvement over the original. Combining two or three statistical potential models further increases success rates, reaching or surpassing several DL approaches. Specifically, HybridSP$_{\mathrm{dk}}$, with summation weights optimized for docking, achieves a 96.5% success rate when the crystal structure is included, only two cases behind the best-performing DL method, RTMScore. This suggests that integrating models based on different interaction distributions benefits near-native pose identification.

**Figure 3:**
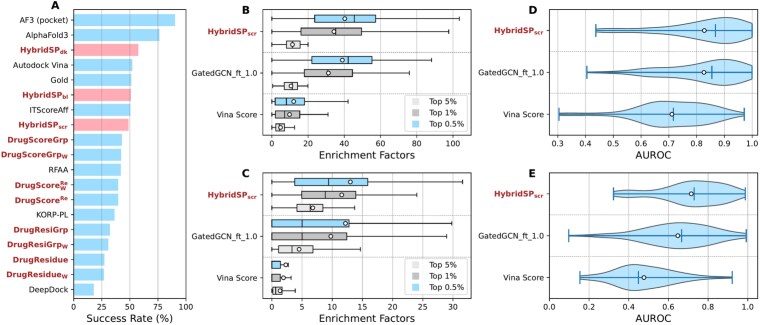
Docking and screening performance across different benchmarks. (A) Docking success rate (top 1) on PoseBusters benchmark set. Screening performance in terms of EFs (F) and success rates (D) on DUD-E dataset. Screening performance in terms of EFs (C) and AUROC (E) on DUD-AD dataset. The average values in (B–E) are labeled as white circles.

We also evaluated docking power on the PoseBusters dataset (Fig. 3A). The workflow involved docking small molecules using AutoDock Vina, followed by post scoring. The results show that among traditional functions, HybridSP$_{\mathrm{dk}}$ performs well, outperforming empirical methods (Gold and Vina) and the DL-based RoseTTAFold All-Atom (without specified pocket positions) [16]. However, a noticeable performance gap remains compared with AlphaFold3, which achieves 90.2% accuracy with a specified pocket. This suggests that diffusion models integrating both evolutionary and structural information are more suitable for such structure modeling tasks, although they may introduce unphysical interactions for mutated pockets and unseen protein–ligand systems [18].

### Screening performance of HybridSP on different benchmarks

For virtual screening, we used the CASF-2016 dataset as a benchmark (Fig. 2C and D). Although most statistical potential-based methods perform well in docking, they show poor screening performance with high false positive rates. This is likely because conventional distance-dependent atom–atom potentials cannot capture diverse ligand–residue interaction patterns. For instance, ITScoreAff and DrugScore$^{\mathrm{Re}}$ perform well in docking but show low EFs in screening. In contrast, DrugResidue and DrugResidue$_{\mathrm{W}}$, which model residue-specific atom–residue distances, achieve better screening results.

We further developed DrugScoreGrp and DrugResGrp by introducing an orientation-dependent atom–residue potential based on geometric relations between a ligand atom and three nearby heavy atoms of a residue. This orientation-aware term improves recognition of both residue-specific and direction-sensitive interactions, resulting in higher EFs and success rates. KORP-PL exhibits a similar advantage. Integrating distance- and orientation-dependent terms, HybridSP achieves accuracy comparable to or better than several DL models. A screening-optimized variant, HybridSP$_{\mathrm{scr}}$, attains the best EF of 29.35.

HybridSP${\mathrm{scr}}$ was also tested on DUD-E and DUD-AD datasets (Fig. 2B–E). On DUD-E, it yields average EFs of 40.27, 34.26, and 11.38 for the top 0.5%, 1%, and 5% of compounds, slightly surpassing GatedGCN_ft_1.0 (38.91, 31.21, and 10.56). Its AUROC score (0.828) is also comparable to GatedGCN_ft_1.0 (0.826). Although all models perform worse on the bias-reduced DUD-AD dataset, HybridSP${\mathrm{scr}}$ remains the most reliable.

**Figure 4:**
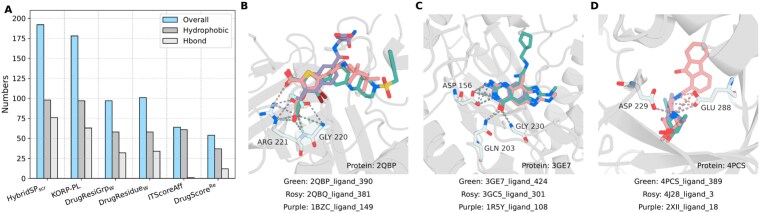
Molecular interactions analysis. (A) The number of common molecular interactions observed among the top five decoys ranked by each scoring function in the CASF-2016 screening benchmark. (B–D) Examples of common hydrogen bond interactions among the top five decoys ranked by HybridSP (only three representative decoys are shown for clarity).

As illustrated in Fig. 1C–F, statistical potentials can capture non-bonded interactions between atoms. We attribute the strong screening performance of HybridSP$_{\mathrm{scr}}$ to its improved ability to accurately identify such interactions. To further investigate this, we evaluated the types of interactions identified by different statistical models. The procedure was as follows: (i) each model was used to score decoys in the CASF-2016 screening benchmark; (ii) for each target, we selected the top five compounds with the best scores along with their best docking poses; (iii) if a specific atom in a target residue was involved in the same type of interaction with all five compounds, we incremented the interaction count by one. For example, in Fig. 4B, the N atom in residue GLY220 acts as a hydrogen bond donor in all five cases, indicating that both the type of interaction and the role of the residue atom must be consistent across all compounds. The types of interactions we analyzed include: hydrophobic interactions, hydrogen bonds, $\mathrm{\pi }$–$\mathrm{\pi }$ stacking, weak hydrogen bonds, salt bridges, amide stacking, cation–$\mathrm{\pi }$ interactions, halogen bonds, and multipolar halogen interactions. The definitions of these interactions follow those proposed by Freitas *et al.* [54].

The number of common identified is shown in Fig. 4A, where the x-axis is sorted from left to right by decreasing EFs. This reveals a clear trend: the stronger the screening power of a statistical potential, the greater its ability to identify shared key interactions. Figure 4B–D presents representative examples from HybridSP. In these cases, the highlighted residues form the same type of interaction (hydrogen bonds) with five top-ranked compounds (for clarity, only three molecules are shown). The compound IDs correspond to decoys from the CASF-2016. Notably, these top-scoring compounds are all true positives for the selected protein target, and they share highly similar local topological features, supporting the rationale behind template-based docking approaches. Furthermore, the figures show that these compounds exhibit strong similarity only at one end, while the other end remains diverse. This observation suggests that our model successfully captures the most critical intermolecular interactions, allowing flexibility in less essential regions.

To further evaluate its generalization ability, we tested HybridSP on cytochrome P450 enzymes, which catalyze nearly 95% of oxidation and reduction reactions and bind structurally diverse ligands [55, 56]. P450s possess flexible binding pockets, making virtual screening particularly challenging. We collected affinity data for eight P450 isoforms from BindingDB and used HybridSP and RTMScore to rescore docked ligands and decoys (Figs S3 and S4). Both methods show reduced accuracy compared with CASF-2016. However, even when no true positive ligands were retrieved, the top-ranked decoys identified by both methods captured key interactions present in the crystal structures. For example, in Figure S3D–F, both HybridSP and RTMScore identified decoy compounds that reproduced the $\mathrm{\pi }$–$\mathrm{\pi }$ stacking observed in the experimental structure (PDB ID: 4I8V). Compared with the crystal ligand conformation, the false-positive compound predicted by HybridSP formed additional polar contacts to the binding pocket. The remaining performance gap may stem from inaccuracies in docking-generated poses and inherent dataset bias, which limit the reliability of the input conformations.

### Evaluation of HybridSP on FEP datasets

**Figure 5:**
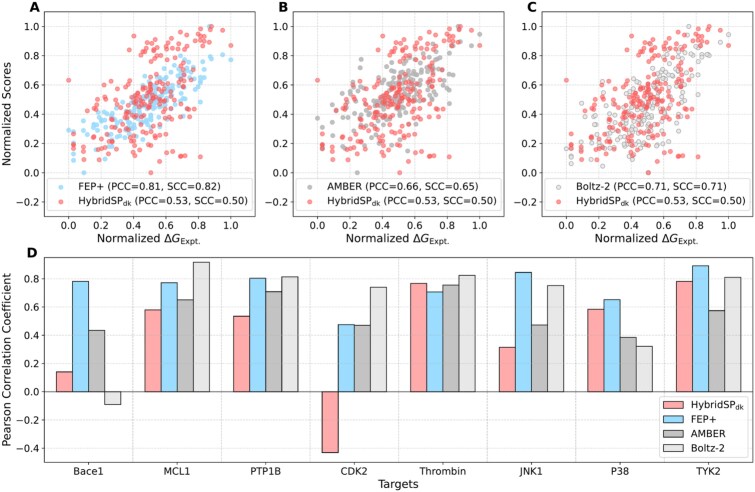
Comparison of overall Pearson and Spearman correlations between different SFs and experimental values on the FEP dataset. Pearson and Spearman correlations between HybridSP and experimental values on the FEP dataset, in reference to (A) FEP+ algorithm, (B) MD simulations based on the AMBER force field and (C) Boltz-2. (D) Pearson correlation coefficients for eight individual targets in the FEP dataset.

FEP is a common method for estimating binding affinities, but it is time-consuming despite its high accuracy [57]. In contrast, statistical potentials like HybridSP are derived from Boltzmann statistics and offer a faster alternative. We evaluated HybridSP’s ability to fit binding free energies on the FEP dataset [36, 37], with the correlation presented in Fig. 5. Since the scores produced by HybridSP do not represent absolute binding free energies ($\Delta G$), we normalized them to a 0–1 scale. When aggregating data across all eight targets, the FEP+ method and AMBER force field-based simulations exhibit better agreement with experimental results than HybridSP. On a per-target cases, FEP+ remains the most accurate method, achieving correlation coefficients >0.7 for most targets. Notably, for the P38, Thrombin, and TYK2 targets, HybridSP$\mathrm{dk}$ outperforms the AMBER-based method in terms of correlation. For the CDK2 target, both FEP methods yields low correlations, while HybridSP$\mathrm{dk}$ showed a negative correlation, which significantly affects its overall performance. It is also worth noting that Boltz-2 [53], designed as a structure prediction method, exhibits good affinity prediction capabilities. Although it shows an unsatisfactory negative correlation on the Bace1 target, it surpasses the other three compared methods on four targets, suggesting that the structures predicted by Boltz-2 are physically and energetically reasonable. These results indicate that while HybridSP exhibits a certain level of accuracy in predicting binding free energies, it still lags behind more rigorous FEP-based approaches.

## Discussion

Our study reveals that even among knowledge-based models, the performance can vary significantly depending on the underlying statistical scheme. Based on these observations, we draw the following three insights.

Incorporating binding affinity into statistical modeling yields more realistic interaction patterns. As shown in Section “Statistical results of DrugResidue$_{\mathrm{W}}$,” weighting statistical potentials by affinity produces more physically meaningful distributions. Statistical potentials estimate energy landscapes from observed contact probabilities, making accurate probability distributions essential. Traditional approaches, however, treat all complexes equally during sampling, ignoring binding strength. High-affinity complexes contain more favorable interactions near energy minima, while low-affinity ones may include suboptimal contacts. Equal weighting thus overrepresents unfavorable interactions, biasing the derived potentials. This is evident in Fig. 1C and D, where affinity-weighted curves suppress high-energy contributions, yielding smoother and more plausible energy profiles. For instance, ITScoreAff integrates affinity during iterative refinement, outperforming its predecessor ITScore in scoring, ranking, and docking. Similarly, in machine learning, affinity-aware training improves generalization. GenScore, with an affinity correction term, surpasses RTMScore in scoring and ranking. IGModel, trained jointly on affinity and RMSD, shows more balanced performance than DeepRMSD, despite both using graph neural networks [24, 25, 30, 39].

Pairwise statistical models with clear physical meaning enhance docking performance. To clarify this observation, we first revisit the physical nature of the docking task. Docking aims to identify the optimal binding pose of a small molecule within a protein pocket [10, 58, 59]. Across docking poses, the ligand–receptor atom types remain unchanged, while interatomic distances vary. Thus, docking can be viewed as adjusting all pairwise distances to minimize the sum of interaction potentials [44, 60]. Accurate pairwise potentials are therefore critical. The clearer their physical interpretation, the better they describe interactions and identify the lowest-energy conformation. For example, DrugScore$^{\mathrm{Re}}$ and ITScoreAff use radial distribution functions to construct pairwise potentials, achieving strong docking performance because such functions fully characterize a system’s thermodynamic state.

In contrast, virtual screening aims to find, among many molecules, those that bind most strongly to a target [10, 61]. Here, ligand–protein atom pairs vary across molecules, so the model must evaluate diverse interaction patterns. Simple distance-dependent atom–atom potentials are insufficient. By modeling atom–residue distances, statistical potentials can capture residue-specific environments and identify ligands best suited for the pocket. Adding orientation enables recognition of multi-atom interactions such as hydrogen bonds and $\mathrm{\pi }$–$\mathrm{\pi }$ stacking, improving screening accuracy. KORP-PL incorporates orientation between ligand atoms and protein backbones [41], enhancing its ability to model direction-sensitive interactions (Fig. 4A). However, its coarse-grained representation limits accurate modeling of pocket microenvironments, reducing both screening and docking performance (Fig. 3A). DL methods, especially graph neural networks, represent proteins and ligands as graphs with physicochemical node and edge features [24, 25, 30]. These frameworks naturally encode local multi-body and orientation-dependent interactions, often achieving higher accuracy than traditional potentials. The relative orientation of atoms and residues is critical for modeling protein interactions. Early structure prediction methods relied primarily on residue–residue distances [62], which could produce symmetric or incorrect folds. trRosetta addressed this by including local orientation parameters [63], leading to its success in CASP13. Both structure prediction and virtual screening depend on accurately modeling local microenvironments, where folding and binding balance enthalpy and entropy.

The performance differences observed between statistical potential models and DL-based scoring functions stem from their fundamentally different modeling paradigms. DL models encode protein–ligand complexes into high-dimensional representations, such as graph-based [24, 25, 30], or contact-based features [39, 64], and rely on large parameter sets to fit complex spatial patterns present in the training data. This high expressive capacity enables them to implicitly capture effects such as relative positioning and solvation-driven preferences, but it can also lead to increased sample specificity and reduced robustness when encountering unseen binding modes or atypical interaction patterns. Recent studies have suggested that AlphaFold3-like co-folding models also prefer to memorize spatial distributions rather than explicit physical interactions, which can limit their interpretability and physical consistency [18].

In contrast, statistical potential models decompose complexes into a smaller set of physically interpretable and degenerate interaction descriptors, such as distance- and orientation-dependent atom–atom or atom–residue statistics. This degeneracy allows structurally diverse complexes to share similar feature representations, improving generalization to unseen systems and preserving physical plausibility. As a result, statistical potentials can remain robust when extrapolating beyond the training distribution, particularly in scenarios where correct interaction patterns are more critical than precise geometric placement. A detailed comparative analysis of representative success and failure cases for HybridSP and RTMScore is provided in Section 3 of the SI (Fig. S2).

It should be noted that not all statistical potential models can achieve DL-level performance. Many earlier approaches are limited by smaller training datasets and under-representative statistical descriptors, which hinder their ability to capture complex protein–ligand interactions. Our results suggest that when statistical potentials are constructed using physically meaningful interaction patterns and supported by sufficiently large and diverse datasets can they approach or match the performance of modern DL models, while retaining advantages in interpretability and robustness.

## Conclusion

In summary, our study introduces a hybrid statistical potential model that combines distance-dependent atom–atom and atom–residue interactions, as well as orientation-dependent atom–residue interactions. This model achieves state-of-the-art performance in both docking and virtual screening tasks, addressing the long-standing challenge of balancing these two objectives in traditional statistical potential frameworks. Although HybridSP still lags behind co-folding models such as AlphaFold3 in structural prediction, it attains accuracy comparable to DL-based scoring functions when evaluating docking poses and even surpasses leading AI models in virtual screening tasks. Moreover, we identified potential distributional biases in the statistical process and proposed an affinity-weighted correction strategy to mitigate them. Our findings highlight that high docking accuracy relies on precise potential descriptions, while effective screening requires the model to accurately capture interactions that are highly dependent on both residue context and spatial orientation. This deeper theoretical understanding will help guide the development of next-generation scoring functions that are not only more accurate but also more interpretable and generalizable across different molecular systems.

**Key Points**

- We introduce HybridSP, a hybrid statistical potential that uniquely integrates distance-dependent atom–atom, atom–residue, and orientation-dependent interactions for protein–ligand binding prediction.
- HybridSP achieves performance competitive with, and even superior to, advanced deep learning models on several benchmarks.
- We demonstrate that docking accuracy relies on precise pairwise potentials, while effective screening requires models that capture residue-specific and orientation-dependent interactions.
- The model incorporates an affinity-weighted statistical scheme to correct for sampling bias in training data, yielding more physically realistic energy profiles.

## Supplementary Material

Supplementary_Materials_bbag088

## Data Availability

HybridSP and other statistical models proposed in this work are available in GitHub public repositories: https://github.com/zelixirSH/HybridSP.git.
