# Supplementary material for: Could statistical potential models achieve comparable or better performance than deep learning models?
Source: Brief Bioinform. 2026 Mar 2;27(2):bbag088. doi: 10.1093/bib/bbag088 (PMC12951076; doi:10.1093/bib/bbag088)
Supplement: Supplementary_Materials_bbag088 [file supplementary_materials_bbag088.pdf]

Supplementary Materials

Zhihao Wang[1], Sheng Wang[2], Jingjing Guo[3], Yuguang Mu[4], Xiangdong Liu[1], Liangzhen Zheng *[2], and Weifeng Li †[1]

[1]*School of Physics, Shandong University, Jinan, China*
[2]*Shanghai Zelixir Biotech Co. Ltd., Shanghai, China*
[3]*Centre for Artificial Intelligence Driven Drug Discovery, Faculty of Applied Sciences, Macao Polytechnic University, Macao SAR, China*
[4]*School of Biological Sciences, Nanyang Technological University, Singapore*

1 Details of newly proposed statistical potential models

1.1 Distance-dependent pairwise atom-atom statistical potential

Distance-dependent pairwise potentials are the most common statistical models, primarily describing the energy associated with the distance r between protein atoms and ligand atoms. Here, we adopt a pairwise statistical approach similar to DrugScore2018, where the potentials are derived from pair distribution functions. But we have refined certain derivation details. The retrained model is named as DrugScore^{Re}. DrugScore^{Re} adopts the same definitions of protein (N_{R}) and ligand (N_{L}) atom types as DrugScore2018 [1], comprising a total of 25 Sybyl atom types. For ligand atoms i and protein atoms j, the pair distribution functions is calculated by the following equations:

$$g_{i,j}(r) = \rho_{i,j}(r)/\rho_{i,j,\mathrm{bulk}}, \tag{1}$$

$$\rho_{i,j}(r) = N_{i,j}(r)/4\pi r^2 \mathrm{d}r, \tag{2}$$

$$\rho_{i,j,\mathrm{bulk}} = \sum_{r=r_0}^{r_c} 3N_{i,j}(r)/(4\pi(r_c^3 - r_0^3)), \tag{3}$$

where $\rho_{i,j}(r)$ denotes the local density within the spherical shell defined by the radius range $(r - \mathrm{d}r/2, r + \mathrm{d}r/2)$, and $\rho_{i,j,\mathrm{bulk}}$ represents the average density of the whole system. The lower limit r_0 for the atom-atom contact range is set at 2 Å to avoid insufficient statistical counts due to excessively short distances, while the upper limit r_c is set at 6 Å to exclude weak interactions at excessively long distances. And the interval dr is 0.1 Å. We normalize the pair distribution function to ensure that $\sum_{r=r_0}^{r_c} g_{i,j}(r) = 1$. As in DrugScore2018, the reference state is defined as the average distribution of all atom pair combinations:

$$g(r) = \frac{\sum_i \sum_j g_{i,j}(r)}{N_{\mathrm{L}} \times N_{\mathrm{R}}} \tag{4}$$

Using the inverse Boltzmann formula, the pseudo free energy difference at a distance r between the atom pair ij and the reference state is given by

$$\Delta W_{i,j}(r) = -k_B T \ln \frac{g_{i,j}(r)}{g(r)} \tag{5}$$

By omitting the dimensional factor $k_B T$ and summing over all ligand atoms, all protein atoms, and all distances, the total energy (total score) of the complex can be obtained:

$$\Delta W = \sum_{i,j,r} \Delta W_{i,j}(r) = -\sum_{i,j,r} \ln \frac{g_{i,j}(r)}{g(r)} \tag{6}$$

*E-mail: zhenglz@zelixir.com
†E-mail: lwf@sdu.edu.cn

In addition to DrugScore^{Re}, we also employed the iterative knowledge-based statistical potential, ITScoreAff, whose fundamental algorithmic principles can be found in the work by Zhao *et al.* [2]. Although ITScoreAff uses an atom type definition different from Sybyl, it groups some atoms from different residues into the same statistical category, thus failing to account for the differences in the residues where the atoms are located. As a result, ITScoreAff still remains the pairwise atom-atom statistical model.

1.2 Distance-dependent pairwise atom-residue statistical potential

Since the same atoms in different residues may result in varying interactions, classifying protein atoms into just 25 Sybyl atom types is not ideal. We classify protein atoms into 100 different types based on residue types. For each residue, two heavy atoms from the backbone and three from the side chain are selected as the preferred atoms. In the case of glycine, which lacks a side chain, all four heavy atoms are considered. Additionally, atoms not defined within protein residues (such as metal atoms, halogens, or certain cofactors, excluding water atoms) are grouped into a single category.

For ligands, the Sybyl atom types are still applicable. With this definition of protein atoms, the number of ligand-protein atom pair contacts is reduced accordingly. As a result, atom pairs related to ligand atoms such as S.O and S.O2 cannot be captured. Therefore, in the distance-dependent pairwise atom-residue statistical model, we only consider 23 ligand atom types. Insufficient contact counts will reduce the accuracy of the model, therefore, we increase the statistical radius interval dr to 0.2 Å.

The derivation of the distance-dependent pairwise atom-residue statistical potential, named as DrugResidue, follows the same procedure as the atom-atom case, with the only differences being that $N_{\mathrm{L}} = 23$ and $N_{\mathrm{R}} = 100$. Residue atom definitions are shown in Table S1.

Table S1: Residue atom definitions of DrugResidue

Residue	Atom	Residue	Atom
ALA	N, CA, CB, O, C	LYS	N, O, CD, CE, NZ
ARG	N, O, CD, NH1, NH2	MET	N, O, CE, CG, SD
ASN	N, O, OD1, CG, ND2	PHE	N, O, CD1, CD2, CZ
ASP	N, O, OD1, OD2, CG	PRO	N, O, CB, CD, CG
CYS	N, CA, O, CB, SG	SER	N, CA, CB, OG, O
GLN	N, O, OE1, CG, NE2	THR	N, O, CB, OG1, CG2
GLU	N, O, OE1, OE2, CG, CD	TRP	N, O, CD2, NE1, CH2
VAL	N, O, CB, CG1, CG2	TYR	N, O, CD1, CD2, OH
HIS	N, O, CB, ND1, NE2, CE1, CD2	GLY	N, CA, O, C
ILE	N, O, CG1, CG2, CD1	OTH	F, CL, BR, I, LI, NA, MG, AL, SI, K, CA,
LEU	N, O, CG, CD1, CD2		CR, MN, FE, CO, CU, ZN, SE, MO, SN

1.3 Orientation-dependent atom-residue statistical potential

We introduce an orientation-dependent statistical potential based on the distances between protein and ligand atoms. Ligand atoms are represented using the Sybyl atom types, consistent with previous models. For each residue, three heavy atoms, mainly from the side chain, are selected to measure their distances to a single ligand atom. The atoms selected in each residue are defined in Table S2. These three distance values encapsulate the geometric relationship between the residue and the ligand atom, providing a representation of interaction orientation. The statistical matrix elements are determined from these distances, enabling the potential to account for directionality in protein-ligand interactions.

The energy between the ligand atom i and a protein residue R is defined as

$$\Delta W(R, r_{ij}, r_{ik}, r_{il}) = -\ln \frac{N_{\mathrm{obs}}(R, r_{ij}, r_{ik}, r_{il})}{N_{\mathrm{ref}}(r_j, r_k, r_l)} \tag{7}$$

r_{ij}, r_{ik}, and r_{il} are the distances between the selected three heavy atoms form residue R and the ligand atom i. The reference state $N_{\mathrm{ref}}(r_j, r_k, r_l)$ is determined by averaging across all atom-residue pairs. We combine the orientation-dependent statistical potential and the distance-dependent statistical potential with a weight ratio of 1:9 to obtain

the overall statistical potential:

$$\Delta W = 0.9 \times \sum_{i \in L} \sum_{j \in P} \sum_{r} \Delta W_{i,j}(r) + 0.1 \times \sum_{i \in L} \sum_{R \in P} \sum_{r_{ij,ik,il}} \Delta W(R, r_{ij}, r_{ik}, r_{il}) \tag{8}$$

When the distance-dependent component is based on an atom-atom statistical potential, the integrated model is designated as DrugScoreGrp. Conversely, when the distance-dependent component is derived from an atom-residue statistical potential, the integrated model is termed DrugResiGrp.

Table S2: Residue atom definitions of orientation term in DrugScoreGrp or DrugResiGrp

Residue	Atom	Residue	Atom
ALA	N, CA, O	LYS	CD, CE, NZ
ARG	CD, NH1, NH2	MET	CE, CG, SD
ASN	OD1, CG, ND2	PHE	CD1, CD2, CZ
ASP	OD1, OD2, CG	PRO	CB, CD, CG
CYS	CA, CB, SG	SER	CB, OG, N
GLN	OE1, CG, NE2	THR	CB, OG1, CG2
GLU	OE1, OE2, CD	TRP	CD2, NE1, CH2
GLY	N, CA, O	TYR	CD1, CD2, OH
HIS	CE1, CD2, NE2	VAL	CB, CG1, CG2
ILE	CG1, CG2, CD1	LEU	CG, CD1, CD2

2 The construction of the P450 dataset

In addition, we constructed a P450 dataset comprising eight protein targets. All available affinity data related to P450 enzymes were retrieved from the BindingDB database [3], using IC_{50} (half-maximal inhibitory concentration) values as the binding affinity. Ligands with affinities below 10 µM were defined as active compounds, while those above this threshold were classified as inactive. To balance the dataset, inactive compounds were supplemented from the ZINC database [4] at an active-to-inactive ratio of 1:200. For each active compound, 200 inactive molecules with similar molecular weights were randomly sampled, and to avoid redundancy, their Morgan fingerprint similarity to the active compound was required to be below 0.95. The initial protein structure was obtained via template-based modeling performed on the SWISS-MODEL server [5]. Following the modeling of the Heme, the entire system was subjected to energy minimization using Z-align for structural refinement [6]. The prepared model was subsequently used for molecular docking with AutoDock Vina [7]. The target structures and lists of active and inactive compounds used for each target are available at `https://github.com/zelixirSH/HybridSP.git`.

3 Results on CASF-2016, Unbias-v2019, and PoseBusters benchmarks

Figure S2 shows the best-ranked docking decoy poses selected by RTMScore and HybridSP for four representative cases, with the crystal structures shown in green. It presents representative examples illustrating complementary strengths and limitations of the statistical potential HybridSP and the deep learning–based scoring function RTMScore. In Figure S2(A–B), HybridSP successfully recovers the correct positioning of the ligand core within the binding pocket but fails to reproduce the orientation of the ligand tail, which in the crystal structure points toward the solvent-exposed region. In contrast, RTMScore correctly identifies this solvent-oriented conformation. This behavior suggests that solvation-related orientation preferences, while not explicitly modeled in statistical potentials that consider only protein–ligand contacts, may be implicitly encoded in deep learning models through patterns learned from training data.

Conversely, Figure S2(C–D) shows cases in which RTMScore selects binding poses that are globally rotated by approximately 180° relative to the crystal structures. Although these poses achieve reasonable shape and positional matching, they fail to reproduce several key polar interactions observed experimentally. HybridSP, on the other hand, correctly recovers these critical interactions, indicating that its scoring is driven by explicit physical interaction patterns rather than purely geometric alignment. These examples highlight that deep learning models may occasionally favor learned spatial configurations at the expense of physical interaction fidelity.

Overall, the cases shown in Figure S2 demonstrate that statistical potentials and deep learning models capture different aspects of protein–ligand recognition. Statistical potentials excel at enforcing physically meaningful interaction

Table S3: Scoring functions performance comparison on CASF-2016

SF	Scoring power (Pearson)	Ranking power (Spearman)	Docking power (w/o native)	Docking power (with native)	Screening power (Top 1% EF)	Screening power (Top 1% SR)
Autodock Vina	0.604	0.528	84.6	90.2	7.7	29.8
DrugResidue	0.361	0.454	80.7	86.7	10.53	26.3
$\text{DrugResidue}_{\text{W}}$	0.389	0.493	80.4	87.0	11.02	24.6
DrugResiGrp	0.424	0.535	83.2	89.5	14.1	33.3
$\text{DrugResiGrp}_{\text{W}}$	0.445	0.535	83.2	87.4	14.68	33.3
$\text{DrugScore}^{\text{Re}}$	0.585	0.635	83.9	88.1	4.91	24.6
$\text{DrugScore}^{\text{Re}}_{\text{W}}$	0.589	0.623	83.9	88.1	4.91	24.6
DrugScore2018	0.602	0.607	79.3	83.5	3.66	15.8
DrugScoreCSD	0.596	0.630	80.4	87.4	5.90	22.8
DrugScoreGrp	0.593	0.619	84.6	89.1	5.96	21.1
$\text{DrugScoreGrp}_{\text{W}}$	0.598	0.614	84.2	88.4	5.96	21.1
GatedGCN_ft_1.0	0.834	0.686	92.6	95.4	23.54	66.7
GT_ft_1.0	0.802	0.684	94.0	96.6	28.12	71.9
$\text{HybridSP}_{\text{bl}}$	0.506	0.614	90.5	95.1	27.95	59.6
$\text{HybridSP}_{\text{dk}}$	0.582	0.658	91.6	96.5	23.32	59.6
$\text{HybridSP}_{\text{scr}}$	0.492	0.591	90.2	95.1	29.35	59.6
IGModel_{pkd}	0.831	0.723	90.9	93.3	19.40	66.7
$\text{ITA+DR}_{\mathbf{W}}$	0.672	0.688	87.7	92.3	11.62	31.6
ITScore	0.583	0.640	77.5	81.4	5.42	11.2
ITScoreAff	0.723	0.668	85.3	89.1	6.30	16.8
KORP-PL	0.447	0.570	85.6	89.1	22.23	42.1
KORP-PL^{W}	0.521	0.561	67.4	78.3	18.5	45.6
$\text{KP+DR}_{\mathbf{W}}$	0.459	0.570	88.8	95.1	29.07	59.6
KP+ITA	0.572	0.665	88.4	93.3	25.66	54.4
RTMScore	0.455	0.529	93.4	97.3	28.0	66.7

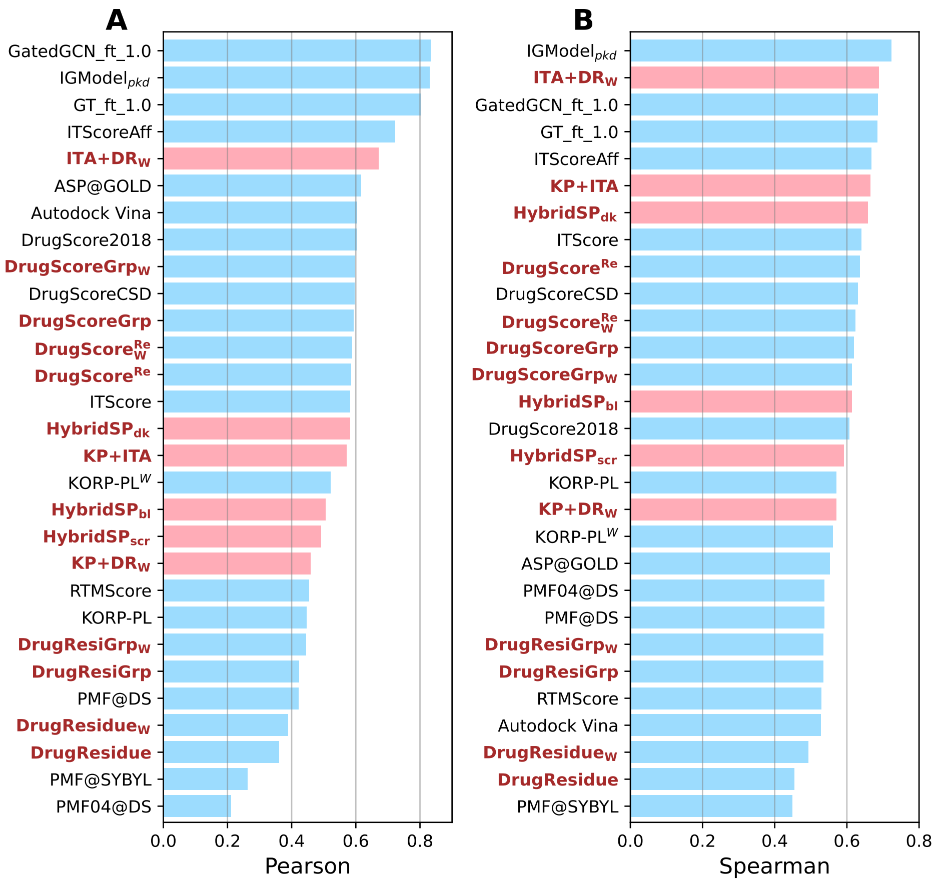

Figure S1: Scoring and ranking performance on CASF-2016 benchmark.

patterns but may struggle with effects mediated by solvent or long-range environmental factors, whereas deep learning models can implicitly account for such effects but may be less sensitive to the precise physical nature of individual interactions.

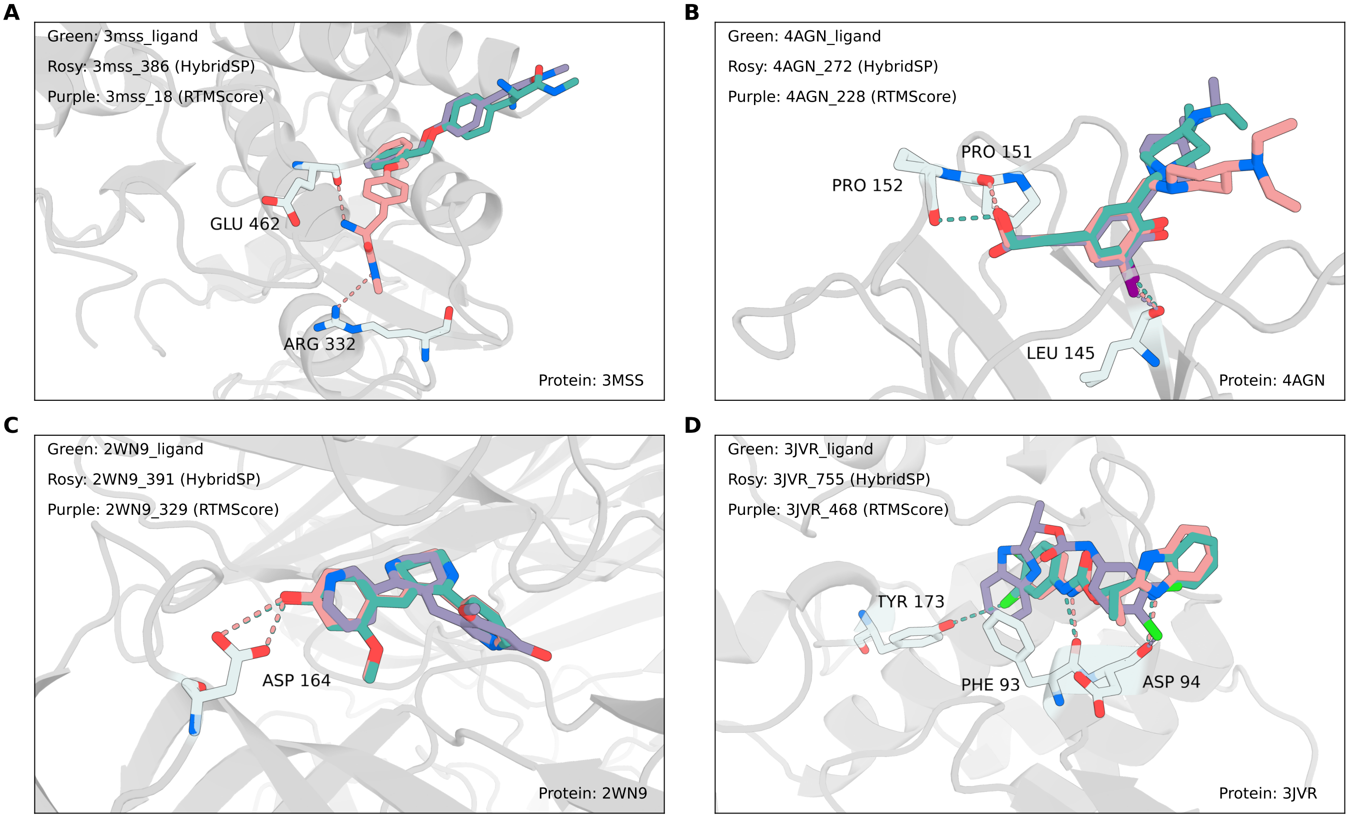

Figure S2: Case studies comparing HybridSP and RTMScore.

Table S4: Docking power comparison on Unbias-v2019 and PoseBusters datasets

SF	Docking power (unbias-v2019)	SF	Docking power (PoseBusters)
zPoseScore	59.9	AF3 (pocket specified)	90.2
IGModel$_{rmsd}$	58.7	AlphaFold3	76.4
IGModel$_{pkd}$	56.2	HybridSP$_{\mathrm{dk}}$	57.5
HybridSP$_{\mathrm{dk}}$	48.6	Autodock Vina	52.3
Autodock Vina	45.3	Gold	51.2
HybridSP$_{\mathrm{bl}}$	45.3	HybridSP$_{\mathrm{bl}}$	50.9
DeepRMSD+Vina	44.1	ITScoreAff	50.5
DrugScoreGrp	42.5	HybridSP$_{\mathrm{scr}}$	48.8
DrugScoreGrp$_{\mathrm{W}}$	42.1	DrugScoreGrp	43.0
HybridSP$_{\mathrm{scr}}$	41.7	DrugScoreGrp$_{\mathrm{W}}$	42.3
ITScoreAff	41.3	RoseTTAFold All-Atom	42.0
DrugScore^{Re}	36.0	DrugScore$^{\mathrm{Re}}_{\mathrm{W}}$	39.7
DrugScore$^{\mathrm{Re}}_{\mathrm{W}}$	35.6	DrugScore^{Re}	39.7
DrugResiGrp	34.4	KORP-PL	36.4
DrugResiGrp$_{\mathrm{W}}$	32.0	DrugResiGrp	32.2
KORP-PL	27.5	DrugResiGrp$_{\mathrm{W}}$	30.8
DrugResidue	27.1	DrugResidue	27.3
DrugResidue$_{\mathrm{W}}$	24.7	DrugResidue$_{\mathrm{W}}$	26.9
DeepRMSD	21.0	DeepDock	17.8

4 Results on DUD-E and DUD-AD benchmarks

Table S5: Performance Comparison on DUD-E Dataset

SF	Top 0.5% EF	Top 1.0% EF	Top 5.0% EF	AUROC
RTMScore	42.47	35.1	10.88	0.83
GatedGCN_ft_1.0	38.91	31.21	10.56	0.824
IGModel$_{pkd}$	27.98	22.15	8.31	0.782
DeepRMSD+Vina	26.5	21.03	8.66	0.806
HybridSP$_{\mathrm{scr}}$	40.267	34.26	11.383	0.828
HybridSP$_{\mathrm{dk}}$	40.885	34.178	11.433	0.826
HybridSP$_{\mathrm{bl}}$	40.426	34.518	11.439	0.828
Vina Score	12.06	9.76	4.79	0.711

Table S6: Performance Comparison on DUD-AD Dataset

SF	Top 0.5% EF	Top 1.0% EF	Top 5.0% EF	AUROC
RTMScore	12.99	10.65	5.1	0.658
GatedGCN_ft_1.0	12.19	9.77	4.52	0.647
IGModel$_{pkd}$	10.1	8.73	4.89	0.686
DeepRMSD+Vina	7.01	6.04	3.4	0.591
HybridSP$_{\mathrm{scr}}$	12.998	11.572	6.778	0.715
HybridSP$_{\mathrm{dk}}$	10.856	9.498	5.952	0.685
HybridSP$_{\mathrm{bl}}$	12.766	11.446	6.733	0.712
Vina Score	2.3	1.92	1.35	0.477

5 Screening results on the P450 dataset

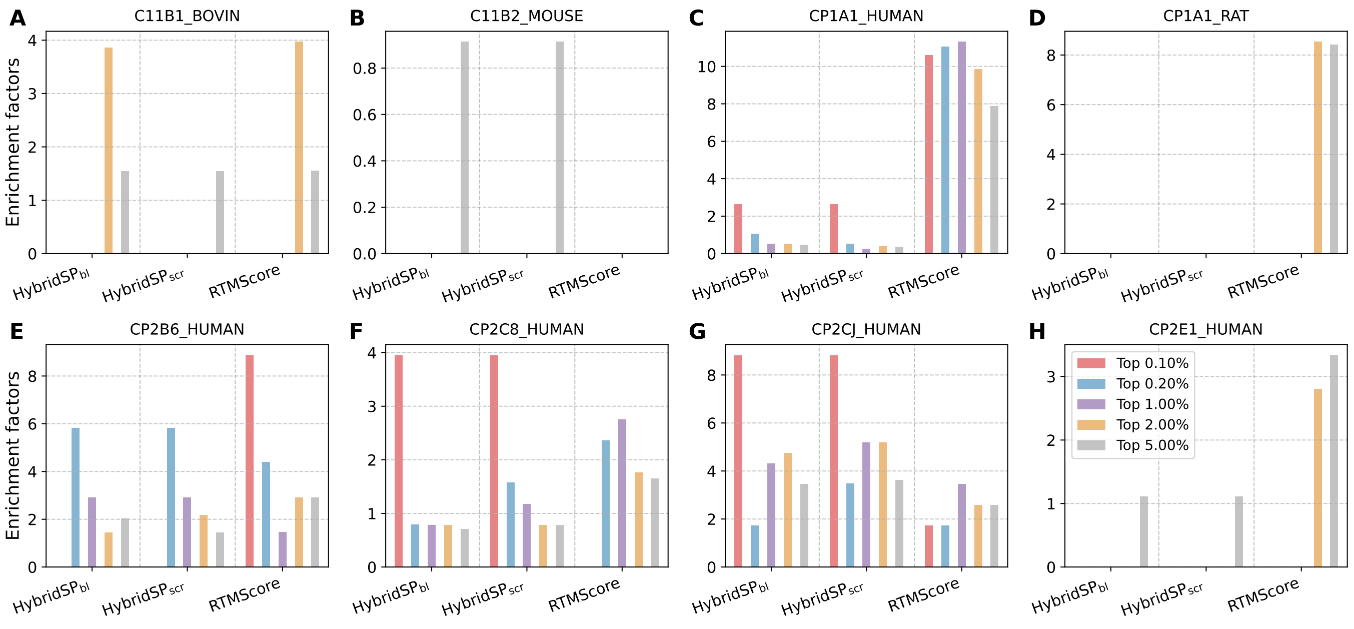

Figure S3: Screening powers of HybridSP and RTMScore on the P450 dataset.

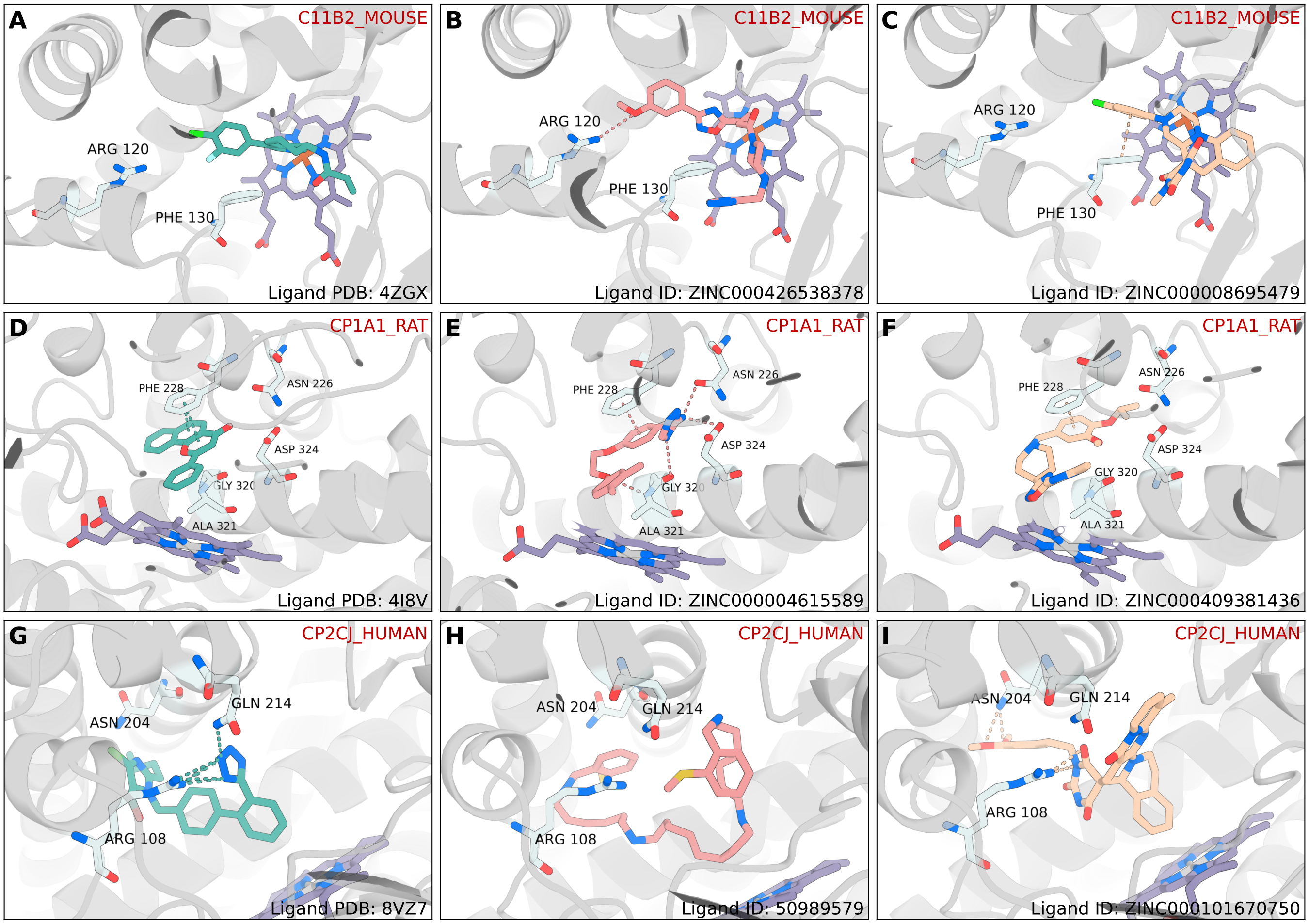

Figure S4: Binding poses of different P450 targets. (A), (D), and (G) show the ligand poses of crystal structures. (B), (E), and (H) show the structures of the best-scoring poses given by HybridSP. (C), (F), and (I) show the structures of the best-scoring poses given by RTMScore.

6 Results on the FEP dataset

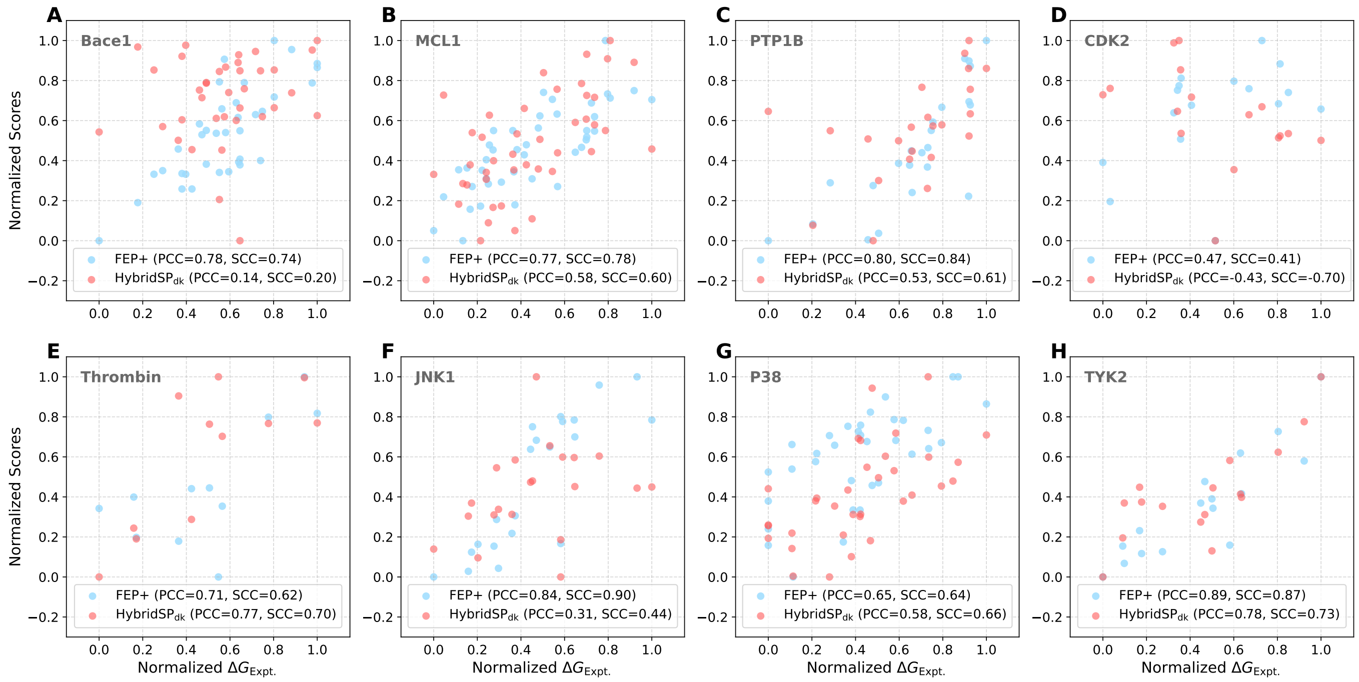

Figure S5: Pearson and Spearman correlations of different targets (A-H) between HybridSP and experimental values on the FEP dataset, in reference to FEP+ algorithm.

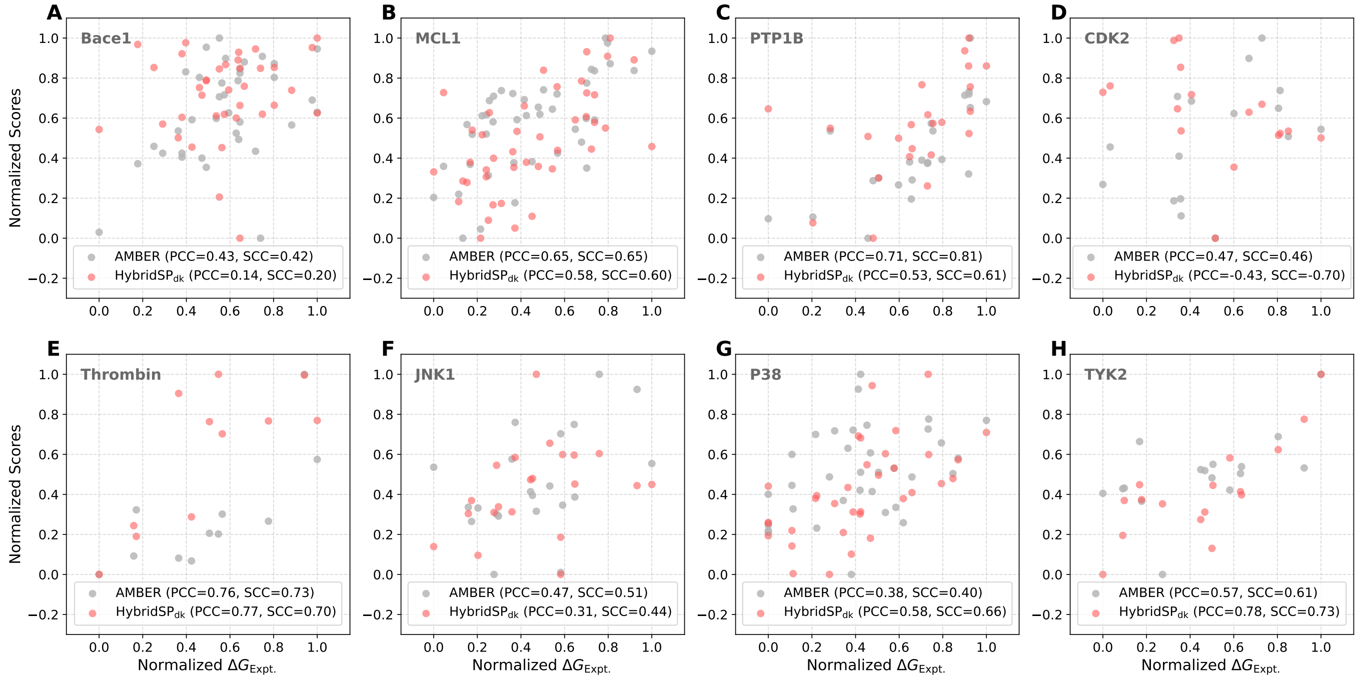

Figure S6: Pearson and Spearman correlations of different targets (A-H) between HybridSP and experimental values on the FEP dataset, in reference to MD simulations based on the AMBER force field

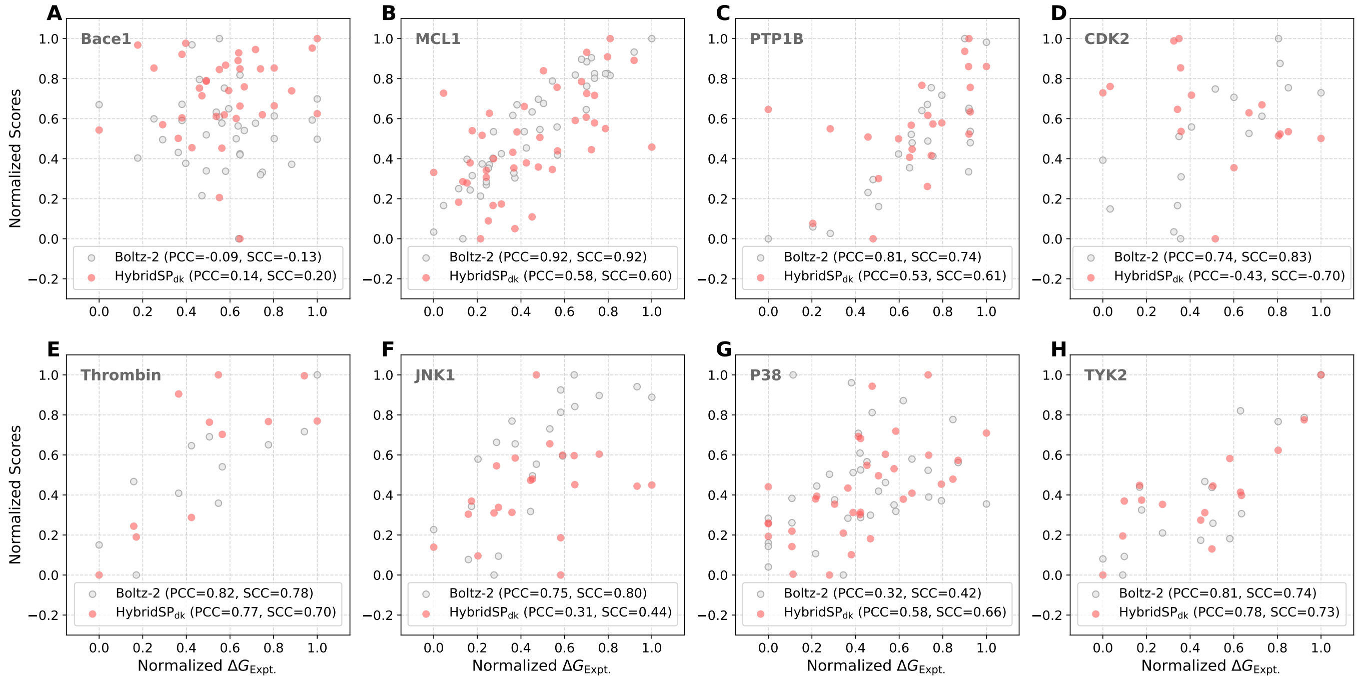

Figure S7: Pearson and Spearman correlations of different targets (A-H) between HybridSP and experimental values on the FEP dataset, in reference to Boltz-2.

7 Computational efficiency

Table S7: Model inference time comparison

Model	Parameters	Time (ms/complex)	
		CPU	GPU
HybridSP	3523	168.0	–
DrugResidue	2300	156.5	–
ITScoreAff	483	12.0	–
KORP-PL	740	4.9	–
IGModel	16.6 M	148.0	117.7
RTMScore	2.7 M	536.9	149.5
GatedGCN	1.1 M	182.1	156.9

In high-throughput virtual screening, computational efficiency is also a critical factor. Therefore, we evaluated the runtime performance of different statistical potential models and DL-based methods under identical conditions. The CASF-2016 docking benchmark was used as the test dataset. All CPU-based evaluations were conducted on a machine equipped with an Intel® Xeon® Silver 4310 CPU (2.10 GHz) and 32 GB of memory, with each model restricted to a single CPU core. For deep learning models, we additionally assessed their performance in a GPU environment using an NVIDIA GeForce RTX 4090 (24 GB), where each model was limited to one CPU core and one GPU, and 32 GB of memory. Each model was executed independently three times, and the average runtime was reported. The results are summarized in Table S2. The results show that KORP-PL and ITScoreAff exhibit a substantial advantage in computational speed, primarily because they are implemented in `C++`. In contrast, $\text{DrugResidue}_{\text{W}}$ and HybridSP, as `Python`-based statistical potential models, incur higher computational costs, which could be mitigated by transitioning to a compiled implementation such as `Cython`. Among the deep learning models, IGModel demonstrates the highest efficiency, slightly outperforming HybridSP in both CPU and GPU modes. With GPU acceleration, RTMScore and GatedGCN can achieve marginally faster runtimes than HybridSP. However, in CPU-only settings, especially for RTMScore, the deep network architecture leads to noticeably reduced efficiency. Given that CPUs are generally more accessible than GPUs, statistical potential models offer a distinct advantage for high-throughput screening, maintaining an attractive balance between computational speed and prediction accuracy.

References

[1] Jonas Dittrich, Denis Schmidt, Christopher Pfleger, and Holger Gohlke. Converging a Knowledge-Based Scoring Function: DrugScore2018. *Journal of Chemical Information and Modeling*, 59(1):509–521, 2019.

[2] Xuejun Zhao, Hao Li, Keqiong Zhang, and Sheng-You Huang. Iterative knowledge-based scoring function for protein–ligand interactions by considering binding affinity information. *The Journal of Physical Chemistry B*, 127(42):9021–9034, 2023.

[3] Tiqing Liu, Yuhmei Lin, Xin Wen, Robert N. Jorissen, and Michael K. Gilson. BindingDB: a web-accessible database of experimentally determined protein–ligand binding affinities. *Nucleic Acids Research*, 35(suppl_1):D198–D201, 2007.

[4] John J. Irwin, Khanh G. Tang, Jennifer Young, Chinzorig Dandarchuluun, Benjamin R. Wong, Munkhzul Khurelbaatar, Yurii S. Moroz, John Mayfield, and Roger A. Sayle. ZINC20—A Free Ultralarge-Scale Chemical Database for Ligand Discovery. *Journal of Chemical Information and Modeling*, 60(12):6065–6073, 2020.

[5] Andrew Waterhouse, Martino Bertoni, Stefan Bienert, Gabriel Studer, Gerardo Tauriello, Rafal Gumienny, Florian T. Heer, Tjaart A P de Beer, Christine Rempfer, Lorenza Bordoli, Rosalba Lepore, and Torsten Schwede. SWISS-MODEL: homology modelling of protein structures and complexes. *Nucleic Acids Research*, 46(W1):W296–W303, 2018.

[6] Zhihao Wang, Fan Zhou, Zechen Wang, Qiuyue Hu, Yong-Qiang Li, Sheng Wang, Yanjie Wei, Liangzhen Zheng, Weifeng Li, and Xiangda Peng. Fully flexible molecular alignment enables accurate ligand structure modeling. *Journal of Chemical Information and Modeling*, 64(15):6205–6215, 2024.

[7] Oleg Trott and Arthur J. Olson. AutoDock Vina: Improving the speed and accuracy of docking with a new scoring function, efficient optimization, and multithreading. *Journal of Computational Chemistry*, 31(2):455–461, 2010.
